# A pathology-attention multi-instance learning framework for multimodal classification of colorectal lesions

**DOI:** 10.3389/fphar.2025.1592950

**Published:** 2025-06-06

**Authors:** Fanglei Fu, Xuemei Zhang, Zhaoxuan Wang, Luxi Xie, Mingxi Fu, Jing Peng, Jianfeng Wu, Zhe Wang, Tian Guan, Yonghong He, Jin-Shun Lin, Lianghui Zhu, Wenbin Dai

**Affiliations:** ^1^ Department of Life and Health, Shenzhen International Graduate School, Tsinghua University, Shenzhen, Guangdong, China; ^2^ Department of Pathology, Liuzhou People’s Hospital Affiliated to Guangxi Medical University, Liuzhou, Guangxi, China; ^3^ Department of Statistics and Data Science, Washington University in St. Louis, St. Louis, MO, United States; ^4^ State Key Laboratory of Cancer Biology, Department of Pathology, Xijing Hospital and School of Basic Medicine, Fourth Military Medical University, Xi’an, China

**Keywords:** multimodal learning, weakly supervised learning, whole slide image classification, pathology attention, colorectal cancer

## Abstract

**Introduction:**

Colorectal cancer is the third most common cancer worldwide, and accurate pathological diagnosis is crucial for clinical intervention and prognosis assessment. Although deep learning has shown promise in classifying whole slide images (WSIs) in digital pathology, existing weakly supervised methods struggle to fully model the multimodal diagnostic process, which involves both visual feature analysis and pathological knowledge. Additionally, staining variability and tissue heterogeneity hinder model generalization.

**Methods:**

We propose a multimodal weakly supervised learning framework named PAT-MIL (Pathology-Attention-MIL), which performs five-class WSI-level classification. The model integrates dynamic attention mechanisms with expert-defined text prototypes. It includes: (1) the construction of pathology knowledge-driven text prototypes for semantic guidance, (2) a refinement strategy that gradually adjusts category centers to adaptively improve prototype distribution, and (3) a loss balancing method that dynamically adjusts training weights based on gradient feedback to optimize both visual clustering and semantic alignment.

**Results:**

PAT-MIL achieves an accuracy of 86.45% (AUC = 0.9624) on an internal five-class dataset, outperforming ABMIL and DSMIL by +2.96% and +2.19%, respectively. On external datasets CRS-2024 and UniToPatho, the model reaches 95.78% and 84.09% accuracy, exceeding the best baselines by 2.22% and 5.68%, respectively.

**Discussion:**

These results demonstrate that PAT-MIL effectively mitigates staining variability and enhances cross-center generalization through the collaborative modeling of visual and textual modalities. It provides a robust solution for colorectal lesion classification without relying on pixel-level annotations, advancing the field of multimodal pathological image analysis.

## 1 Introduction

Colorectal cancer (CRC) is the third most common type of cancer globally and the second leading cause of cancer-related deaths (Bray et al., 2024). The classification of colorectal epithelial lesions generally includes the following categories: non-tumor lesions (e.g., inflammatory polyps), benign epithelial tumors and precursors (e.g., hyperplastic polyps, adenomatous polyps with low-grade or high-grade dysplasia), and malignant epithelial tumors (e.g., colorectal adenocarcinoma and neuroendocrine neoplasms) (World Health Organization, 2019). These different lesion grades reflect varying risks of malignancy and guide corresponding intervention strategies (Wei et al., 2020). Hyperplastic polyps are common benign epithelial lesions with a low risk of malignant transformation, but they still require regular monitoring. Tubular adenoma is a frequently observed subtype of adenomatous polyps, and due to its higher potential for malignancy, early removal is typically recommended. This is particularly important as tubular adenomas may progress to malignant lesions if not timely intervened (Torlakovic et al., 2003). High-grade intraepithelial neoplasia is a precancerous condition that exhibits significant cytological abnormalities and a high tendency for malignancy, thus necessitating proactive intervention. Once epithelial lesions progress to the adenocarcinoma stage, it indicates that the lesion has developed into an uncontrolled malignant proliferative state, usually requiring comprehensive treatment approaches such as surgery and chemotherapy (Yengec-Tasdemir et al., 2023). Therefore, accurately distinguishing lesion categories during diagnosis is of critical importance.

Deep learning has shown great potential in recognizing disease-specific histomorphological patterns. It has also been widely applied in automated biomarker detection (Niazi et al., 2019; Song et al., 2023; Li et al., 2022; Perez-Lopez et al., 2024). Recent studies have shown that deep learning techniques can classify conventional H&E stained, formalin-fixed, paraffin-embedded digital WSI of colorectal cancer into microsatellite stable and microsatellite unstable categories, sometimes outperforming board-certified pathologists (Yamashita et al., 2021; Wagner et al., 2023). Furthermore, the use of pre-trained models, which are widely applied in the field of pathology, (Chen et al., 2024; Vorontsov et al., 2023; Ding et al., 2024; Xu et al., 2024) has significantly enhanced the capability of models to extract morphological features. However, due to the massive scale of WSI data and the complexity of professional interpretation, manually annotating pixel-level details is extremely challenging. To address this issue, researchers have developed weakly supervised learning algorithms (Ilse et al., 2018; Lu et al., 2021; Li et al., 2024; Li et al., 2021; Bontempo et al., 2023; Chikontwe et al., 2024, Shao et al., 2021) that enable models to be trained using only slide-level labels. While this approach alleviates some of the challenges associated with data annotation, there remains a gap between how models operate in both traditional supervised and weakly supervised learning and actual clinical practice. In standard clinical diagnostic processes, pathologists rely on extensive prior pathological knowledge combined with identified tumor regions to make comprehensive judgments. Therefore, an important research direction is to better simulate the diagnostic process of pathologists and further integrate deep learning with clinical practice (Shi et al., 2024; Tang et al., 2023; Liu et al., 2024; Yu et al., 2023; Zhang et al., 2025).

Iizuka’s team (Iizuka et al., 2020) proposed an automatic classification method for colorectal polyps based on deep convolutional neural networks. They used the Inception-v3 network for patch-level classification and employed recurrent convolutional neural networks (RCNNs) for WSI prediction. However, this method was only applied to a binary classification task distinguishing between adenocarcinoma and adenoma, achieving AUCs of 0.96 and 0.99, respectively. On the other hand, Wei et al. (2020) utilized deep residual networks (ResNet) to classify polyps as either adenomatous or serrated. They compared the model’s predictions with diagnoses from local pathologists, achieving an accuracy of 93.5%. Recently, Perlo and colleagues proposed using ResNet for grading dysplasia in colorectal polyps (Barbano et al., 2021). They considered six different types of polyps and provided WSI-level predictions. Using ResNet-18 on 600 µm slides, they achieved a 70% diagnostic accuracy at the WSI level. More recently, the team led by Yengec-Tasdemir proposed combining Sup-Con and BiT for a three-class classification task of colorectal polyps, achieving an accuracy of 86.2% on their custom dataset and 70.1% on the UnitoPatho dataset (Yengec-Tasdemir et al., 2024).

In recent years, methods combining contrastive learning with text supervision have gradually emerged in the field of pathology image analysis (Stacke et al., 2020; Lu et al., 2019; Ciga et al., 2022; Wu et al., 2022; Boserup and Selvan, 2022; Ke et al., 2021). The CLIP model links images with corresponding textual descriptions through contrastive learning, enabling the model not only to recognize image features but also to understand diagnosis-related textual information. The PLIP model (Huang et al., 2023), fine-tuned based on CLIP (Ciga et al., 2022; Radford et al., 2021), further integrates pathological text labels with WSI data, effectively localizing diagnosis-related regions and improving data efficiency. Meanwhile, the CONCH model leverages pre-training on over 1.17 million image-text pairs for unrelated tasks, demonstrating exceptional multimodal understanding and transfer capabilities. In 14 pathology benchmarks, CONCH (Lu et al., 2024) achieved leading performance in tasks such as classification, segmentation, description generation, and image retrieval, and can adapt to various downstream tasks with minimal additional fine-tuning, showcasing its broad application potential.

We propose a multimodal deep learning framework for a five-class classification task in colorectal cancer pathology, which integrates a dynamic attention mechanism with semantic guidance from expert-defined text prototypes. By focusing on diagnostic-relevant regions through an attention-based module, our method effectively suppresses noise from irrelevant areas. Meanwhile, the text-driven prototype optimization mechanism enhances the alignment between visual and semantic features, mitigating the impact of data variations such as staining differences. Additionally, for the task specific to colorectal pathology, we employed various pre-trained image feature extractors and selected the one with the best performance. This collaboration between visual and text modalities enables the model to generate robust WSI-level representations, demonstrating exceptional performance and adaptability across diverse datasets and complex cancer subtypes.

## 2 Experimental setup and data

The WSIs required for the development and evaluation of our method were collected from patients undergoing colorectal cancer screening at three medical centers: Xijing Hospital, Liuzhou People’s Hospital, and Zhongnan Hospital. Our team of expert pathologists collaborated to annotate different types of colorectal pathological morphologies in these images. We used these annotations as the reference standard for training and testing our deep learning method to classify colorectal pathology across whole slide images.

In this project, the proposed method requires data collected from patients who underwent colorectal cancer screening at our partner medical center since January 2020. Through collaboration with various pathology centers, we accumulated a total of 5,062 pathology WSIs, including both biopsy and surgical samples. The WSIs were scanned and digitally stored using the SQS 1000 or SQS-2000 scanners provided by Shenzhen Shengqiang Technology Co., Ltd., with an objective magnification of 20x. Images that remained unclear after multiple scans were excluded. Our training dataset comprises 1756 H&E-stained whole slide images. In this study, we employed five-fold cross-validation for model training and validation, ensuring balanced representation of each class in the training, validation, and test sets through stratified splitting. Specifically, 1,263 samples were selected for training, 141 for validation, and 352 for testing. Additionally, 1,163 external cases were used as an external test set.

The WSIs do not overlap, and each WSI belongs to a different patient or colonoscopy procedure. As shown in Table 1, our histological imaging dataset includes five types of colorectal H&E stained WSIs: normal (non-tumor lesions), hyperplastic polyp, adenoma, high-grade intraepithelial neoplasia, and adenocarcinoma. These five categories cover all stages of colorectal pathological development and encompass all types in the WHO classification of colorectal tumors.

**TABLE 1:** Distribution of the internal dataset.

	Normal	HP	Adenoma	HGIN	Carcinoma	Total
Training	385	101	287	128	362	1,263
Validation	43	11	32	14	41	141
Test	108	29	79	36	100	352
Total	536	141	398	178	503	1,756

The high-resolution histological images of colorectal polyp samples are large. Most regions in non-normal colorectal WSIs are normal, with only a small portion actually related to colorectal polyps or tumors. During the data annotation process, to ensure accuracy, we invited 2-3 experts with over 20 years of pathology experience to annotate the slides. They combined clinical information, imaging data, morphological information, and immunohistochemical results to reach the final annotation. In case of disagreements, an additional expert with over 20 years of clinical pathology experience was invited to review the slides. If a consensus was reached among the majority of experts, the case was included in subsequent experiments; otherwise, it was excluded.

**FIGURE 1:**
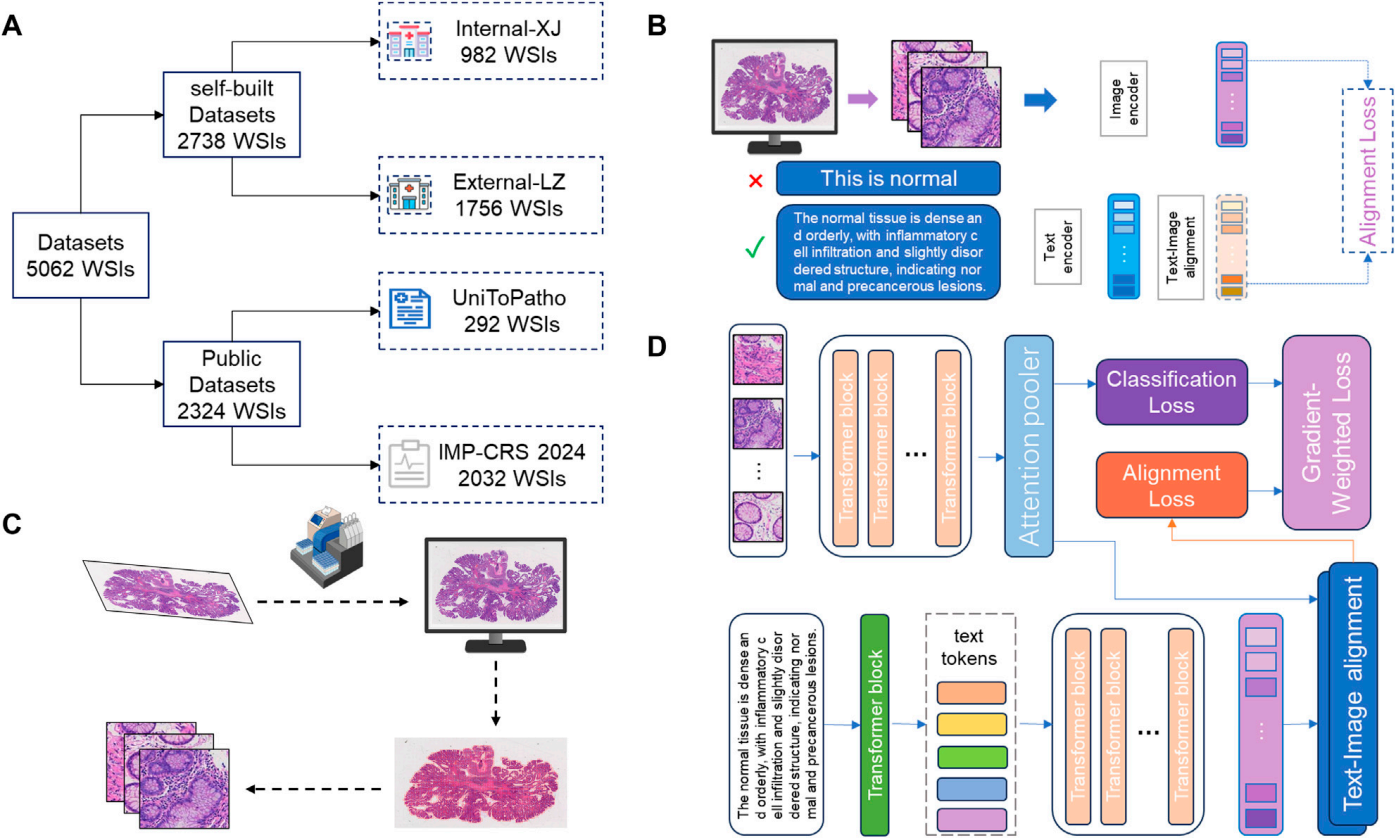
Workflow of the deep learning model. **(A)** Data Source and Division: This study utilized 5062 H&E stained WSIs from four different centers. Data from Liuzhou Hospital served as the internal dataset for model training, while data from Xijing Hospital was used as an external test set. Additionally, two publicly available datasets were used to construct extra external datasets to evaluate the model’s generalization capability. **(B)** Construction and Optimization of Encoder: The image encoder and text encoder used in the model were trained through contrastive learning on large-scale pathology image-text pairs. The text content was optimized and adjusted by pathology experts to capture more robust pathological representations, thereby enhancing the model’s performance in practical applications. **(C)** Data Preprocessing: After digitizing the slides, the tissue regions were segmented, and the entire WSI was divided into multiple patches to facilitate subsequent feature extraction and analysis. **(D)** Model Computation Process: The core computation process of the deep learning model is divided into three stages: (1) Slide-level feature generation and prediction based on images; (2) Slide-level feature generation and prediction based on text; (3) Loss calculation dynamically adjusted according to the loss gradient to balance the contributions of image and text features, thereby optimizing the final classification performance.

As shown in Figure 1, in this study, we also utilized two publicly available pathology datasets. The first is the UniToPatho (Barbano et al., 2021) dataset, which includes 292 WSIs acquired at ×20 magnification (0.4415 μm/px) using a Hamamatsu Nanozoomer S210 scanner. Each WSI is from a different patient. These images have been annotated by pathology experts into six categories: Normal tissue (NORM), Hyperplastic Polyp (HP), Tubular Adenoma with High-Grade Dysplasia (TA.HG), Tubular Adenoma with Low-Grade Dysplasia (TA.LG), Tubulovillous Adenoma with High-Grade Dysplasia (TVA.HG), and Tubulovillous Adenoma with Low-Grade Dysplasia (TVA.LG). The second dataset is the IMP-CRS 2024 dataset (Oliveira et al., 2021; Neto et al., 2022; Neto et al., 2024), which consists of 5,333 colorectal biopsy and polypectomy WSIs from the data archive of the IMP Diagnostics Laboratory in Portugal, with 2032 WSIs used in this study. These WSIs were digitized using two Leica GT450 WSI scanners at ×40 magnification and annotated into three categories: Non-neoplastic lesions, Low-Grade Lesions (conventional adenomas with low-grade dysplasia), and High-Grade Lesions (conventional adenomas with high-grade dysplasia and intramucosal adenocarcinoma).

## 3 Methods and architecture

### 3.1 Overall framework

The method of aggregating WSI feature representations through attention modules to learn robust representations for medical image visual tasks has achieved significant success. Meanwhile, in the field of natural images, research has shown (Ciga et al., 2022) that textual information can significantly enhance the performance of image-based models. However, previous pathology studies have typically relied on paired WSI and diagnostic report content, which requires large annotated datasets to train a robust foundational model, limiting the model’s application in data-scarce scenarios.

### 3.2 Construction of text prototypes

Our research takes a different approach: by extracting the expert diagnostic knowledge of pathologists and encapsulating these experiences into cancer-specific descriptions, we further construct a set of pathology report prototypes associated with different categories. These prototypes are manually curated by expert pathologists based on real diagnostic expressions and key morphological features, offering precise, interpretable semantic anchors that align with clinically meaningful pathology categories. These pathology report prototypes (hereafter referred to as “text prototypes”) serve as semantic guidance, enabling slide-level features to align more closely with category semantics, thereby effectively alleviating variability in visual representations such as staining intensity and tissue morphology. This innovative strategy, combining pathology reports with attention mechanisms, not only compensates for the data dependency shortcomings of traditional methods but also further enhances the model’s generalization ability and diagnostic performance in pathological tasks.

### 3.3 Dynamic prototype refinement

To ensure more reasonable visual clustering, we designed a dynamic fine-tuning module for pathology reports and a matching module for pathology reports and pathology images. Specifically, this module dynamically adjusts the text prototypes based on the distribution information of instance features within the WSI. Through this mechanism, the text prototypes can not only express the global semantic information of the categories but also gradually adapt to the feature distribution of specific slides, forming clustering centers that better align with the actual data. This process effectively bridges the gap between the textual semantic space and the visual feature space, providing more precise category representations for subsequent classification tasks. Dynamic fine-tuning updates the prototypes through feature and category matching weights:

$$p_{k}^{\left(t+1\right)}=\alpha p_{k}^{\left(t\right)}+\left(1-\alpha \right)\cdot \frac{1}{\left|S_{k}\right|}\sum_{i\in S_{k}}z_{i}$$

Where the initial pathology report is p_k_^(0)^, S_k_ is the sample set of class k, α is the smoothing parameter that controls the update strength, and z_i_ represents the sample features.

For a given class, the text-guided prototype (p_k_) and the slide-level feature z_i_, the pathology report supervision loss is defined as:

$$L_{\text{text}}=-\frac{1}{N}\sum_{i=1}^{N}\sum_{k=1}^{K}y_{ik}\log\left(\frac{\exp\left(\cos\left(z_{i},p_{k}\right)\right)}{\sum_{j=1}^{K}\exp\left(\cos\left(z_{i},p_{j}\right)\right)}\right)$$

Among them, $\cos\left(z_{i},p_{k}\right)=\frac{z_{i}\cdot p_{k}}{\left\|z_{i}\right\|\left\|p_{k}\right\|}$ , and y_ik_ is the class label of sample i.

### 3.4 Attention-based instance aggregation

Our model employs an attention-based module to perform weighted aggregation of instance features from WSI. This module learns the importance weights of instances, adaptively focusing on the regions most relevant to the classification task, enabling the model to effectively extract global representations from large-scale unstructured data.

For a WSI, $X=\left\{x1,x2,\ldots ,xn\right\}$ , where $x_{i}$ represents instance features, the attention module performs weighted aggregation using the weight $a_{i}$ :

$$a_{i}=\frac{\exp\left(h\left(x_{i};\theta \right)\right)}{\sum_{j=1}^{n}\exp\left(h\left(x_{j};\theta \right)\right)},z=\sum_{i=1}^{N}a_{i}x_{i}$$

Among them, $h\left(x_{i};\theta \right)$ is the attention scoring function used to compute the weights, and z represents the slide-level features.

By computing the weighted slide-level features, the visual supervision loss is defined as:

$$L_{\text{vis}}=-\sum_{k=1}^{C}y_{k}\log\frac{\exp\left(w_{k}^{⊤}z+b_{k}\right)}{\sum_{j=1}^{C}\exp\left(w_{j}^{⊤}z+b_{j}\right)}$$

Where W is the weight matrix of the visual classification module, and b is the bias vector.

### 3.5 Dual-loss dynamic weighting strategy

In order to effectively integrate text prototypes and visual clustering prototypes, we propose a dual-loss dynamic weighting method based on gradient magnitude. Specifically, during the training process, the model calculates the gradients of the two types of losses in real-time and dynamically adjusts their weights according to their relative magnitudes. This approach achieves a balance between semantic consistency and visual feature clustering. The dynamic adjustment mechanism ensures effective synergy between text and image information sources, providing a new direction for model optimization.

Given the loss functions $L_{\text{text}}$ and $L_{\text{vis}}$ , the formula for dynamically adjusting the coefficient based on the gradient is:

$$\lambda_{\text{text}}=\frac{\left\|\nabla L_{\text{text}}\right\|}{\left\|\nabla L_{\text{text}}\right\|+\left\|\nabla L_{\text{vis}}\right\|},\lambda_{\text{vis}}=\frac{\left\|\nabla L_{\text{vis}}\right\|}{\left\|\nabla L_{\text{text}}\right\|+\left\|\nabla L_{\text{vis}}\right\|}$$

The final computed total weight is:

$$L=\lambda_{\text{text}}L_{\text{text}}+\lambda_{\text{vis}}L_{\text{vis}}$$

The final approach not only eliminates the reliance on paired data but also significantly enhances the model’s robustness and generalization capabilities. It offers a novel perspective for the classification of pathological WSIs with staining inconsistencies and significant feature variations. This integrated method, based on multimodal information from text and images, demonstrates its potential in cancer pathology classification and provides important insights for a broader range of medical image analysis tasks.

## 4 Results

In the first part of our study, we focused on selecting the most suitable image feature extractor to provide robust feature representations for subsequent experiments. To achieve this, we evaluated several pre-trained large models, testing their performance on both an internal five-classification dataset and the publicly available CRC-2024 dataset. For the internal dataset, we employed a 5-fold cross-validation approach, while for the CRC-2024 dataset, we conducted evaluations based on the test set division provided by the official source.

The experimental results demonstrate that the Virchow model excels in key metrics such as accuracy, F1-score, and AUC. Notably, on the CRC-2024 dataset, its AUC reached an impressive 0.9949, showcasing its superior feature extraction capability. In comparison, although other models come close to Virchow in certain metrics, their overall performance is slightly inferior. Therefore, our experiments confirm the advantage of the Virchow model in colorectal pathology image classification tasks. Based on this, we have chosen Virchow as the preferred feature extractor for subsequent experiments to ensure that the model obtains high-quality feature representations, thereby enhancing overall performance.

**FIGURE 2:**
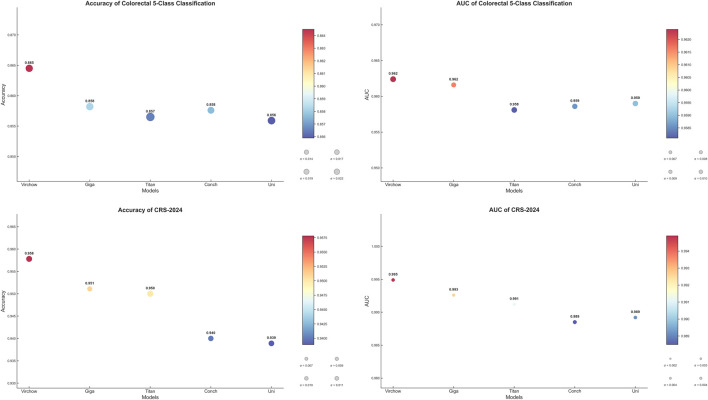
Selection of baseline feature extractors.

The area under the Receiver Operating Characteristic (ROC) curve (AUC) is a key metric in medical image classification, providing a comprehensive evaluation of model performance across different decision thresholds. To facilitate an intuitive comparison and selection of baseline feature extractors, we visualized the accuracy and AUC scores of different models on various datasets in Figure 2. This graphical representation allows us to clearly and directly assess the relative advantages of various feature extraction methods for our specific medical imaging task.

Based on the above considerations, we ultimately selected Virchow as the baseline model to provide the best feature representation for subsequent experiments. This choice not only lays a solid foundation for our research but also enhances the model’s robustness to staining variations and rare lesion types, making it more applicable to real-world clinical settings.

To comprehensively evaluate the performance of our proposed text-supervised image classification model, we conducted comparative experiments with other classic weakly supervised models in the multiple instance learning domain. These models include ABMIL, CLAM, WIKG, DS, and TRANS, which have shown excellent performance in weakly supervised classification tasks but have not fully utilized the supervisory signals from text information. Our experiments employed the same feature extractor (Virchow) as the baseline model and were trained and evaluated under a unified experimental setup. To thoroughly assess the performance of each model, we compared key metrics such as classification accuracy, AUC, and F1 score.

As shown in Table 2, our text-supervised model outperformed other weakly supervised models across all datasets and most metrics. On the colorectal 5-class dataset, our model achieved an accuracy of 86.45% and an AUC of 0.9624, representing improvements of 2.19% and 0.0043, respectively, compared to the best baseline model DSMIL. On the CRS-2024 dataset, our model performed exceptionally well, achieving an accuracy of 95.78% and an AUC of 0.9949, surpassing all other baseline models. On the UNITOPATHO dataset, our model also demonstrated excellent performance, with an accuracy of 84.09% and an AUC of 0.9568, representing improvements of 5.68% and 0.0137, respectively, compared to the best baseline model CLAM_SB. These results fully demonstrate the stability and superiority of our proposed text-supervised model across multiple datasets.

**TABLE 2:** Comparison of the proposed method and other methods.

Dataset	Internal-XJ		CRS-2024		UNITOPATHO	
	ACC	AUC	ACC	AUC	ACC	AUC
ABMIL	0.8349	0.9578	0.9256	0.9861	0.7614	0.9254
CLAM_MB	0.8343	0.9587	0.9356	0.992	0.7500	0.9405
CLAM_SB	0.8383	0.9594	0.9333	0.9909	0.7841	0.9431
DSMIL	0.8426	0.9581	0.9167	0.9857	0.7143	0.8582
TRANSMIL	0.8155	0.9296	0.9100	0.9802	0.6818	0.8812
WIKGMIL	0.8013	0.9424	0.9067	0.9798	0.7727	0.9469
OURS	**0.8645**	**0.9624**	**0.9578**	**0.9949**	**0.8409**	**0.9568**

Bold values indicate the best model performance among the compared models.

Compared to models like ABMIL, our text-supervised model can better leverage the guiding role of textual features, significantly enhancing the expression capability of cross-modal features. Although CLAM and WIKG perform well in the weak supervision domain, their low reliance on text when handling multimodal data leads to suboptimal performance in diverse tasks. Our model effectively integrates textual information, not only improving classification accuracy but also enhancing the robustness and generalization ability of the model. This advantage is particularly evident in complex medical image classification tasks, with a notable improvement on the UniToPatho dataset, highlighting the exceptional performance of our model in handling diverse pathological images. This provides new insights and methods for future multimodal medical image analysis, demonstrating the tremendous potential of text supervision in enhancing medical image classification performance. This may assist pathologists in interpreting complex or ambiguous lesions by providing more consistent, semantically informed predictions.

**FIGURE 3:**
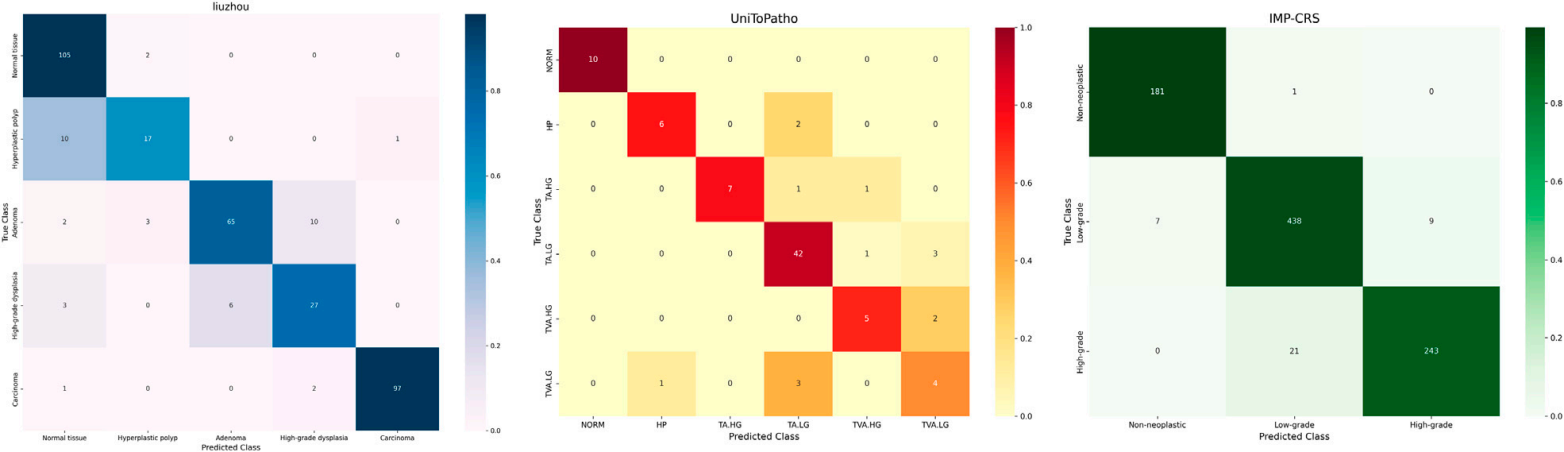
Confusion matrices for different datasets.

The classification confusion matrices in Figure 3 provide us with an in-depth understanding of the performance of the proposed method. These matrices show the distribution of predicted classes relative to the true labels on each dataset. High values along the diagonal of the matrix indicate accurate classification results, while values off the diagonal represent misclassifications. Our method demonstrates improved classification performance with fewer misclassification instances. In the IMP-CRS dataset task, the model excels in distinguishing between non-tumorous lesions, low-grade lesions, and high-grade lesions, with only a few misclassifications between low-grade and high-grade lesions. In our self-constructed dataset task, where categories are further refined, the model achieves high classification accuracy while effectively distinguishing between cancerous and non-cancerous cases, reaching 99.6% specificity and 99.0% precision. In the more granular UniToPatho dataset task, although there is room for improvement in distinguishing certain similar lesion types (such as hyperplastic polyps and tubulovillous adenomas with low-grade dysplasia), the model overall achieves high classification accuracy. These results not only validate the effectiveness of our method but also provide clear directions for further model optimization.

**FIGURE 4:**
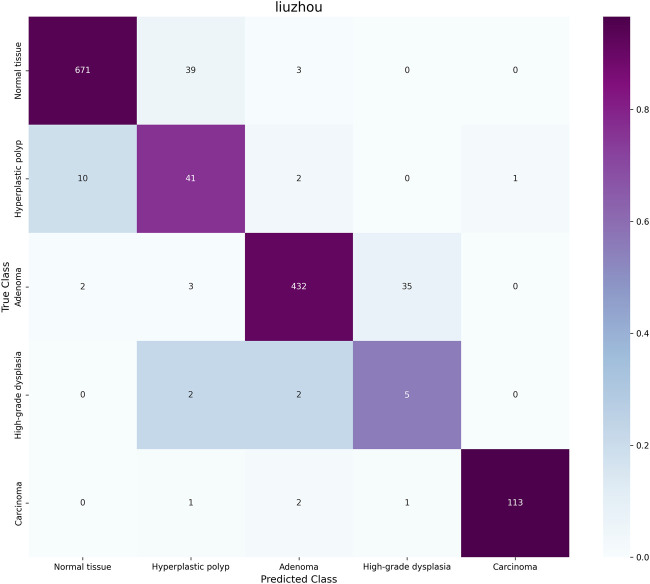
Confusion matrix for the external test set.

On the external test set, the distribution of samples for each class is as follows: normal has 713 samples; hyperplastic polyp has 54 samples; adenoma has 472 samples; high-grade intraepithelial neoplasia has 9 samples; and adenocarcinoma has 117 samples. As shown in Figure 4, our model, PAT-MIL, achieved an overall accuracy of 86.39% on this test set, which is consistent with the results obtained on our internal dataset. This result indicates that the trained model demonstrates good generalization ability across data from different pathology centers, maintaining consistent discriminative performance among different categories. It validates the robustness of the model with diverse data sources.

**FIGURE 5:**
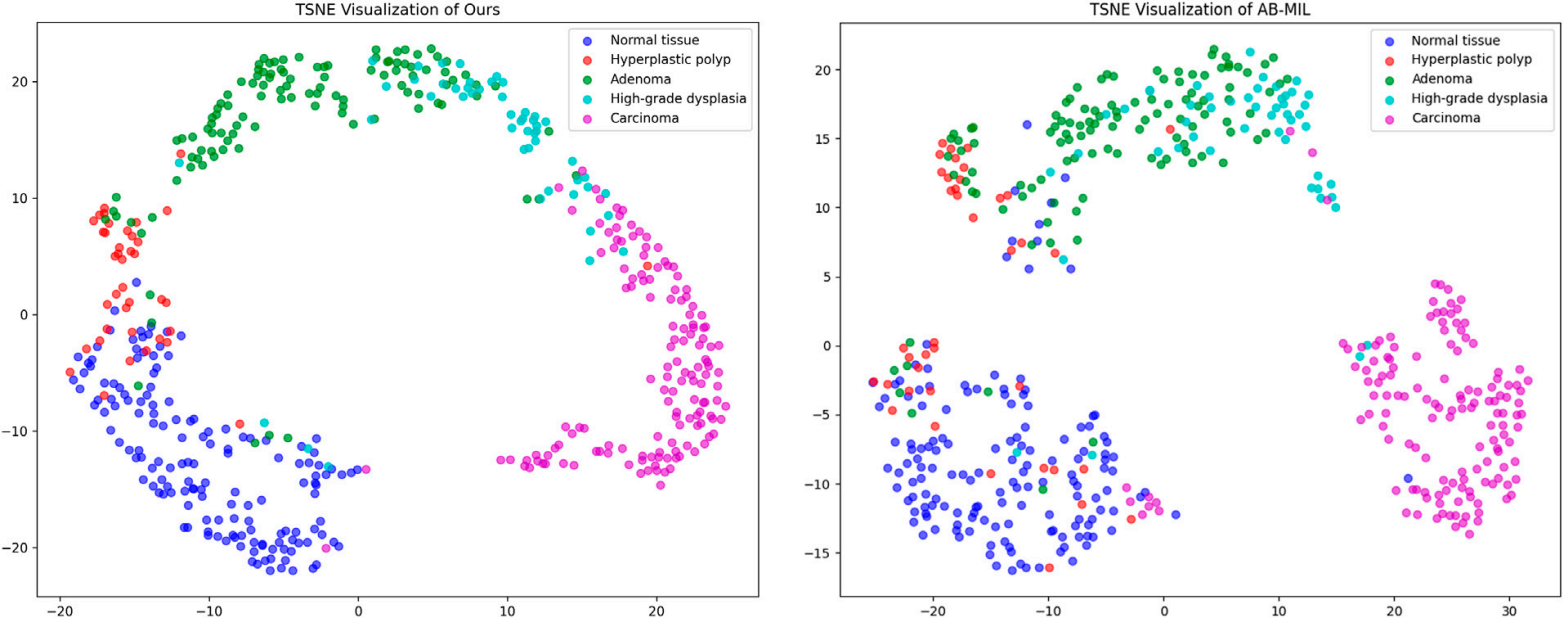
t-SNE Dimensionality Reduction Plot–Left: Proposed Method; Right: AB-MIL.

To further intuitively demonstrate the differences in feature representation between our proposed method and other approaches, we utilize t-SNE (t-distributed stochastic neighbor embedding) to perform dimensionality reduction and visualization of the high-dimensional features extracted by the models. Figure 5 presents the dimensionality reduction results of our method, ABMIL, and our own method on the self-constructed five-class dataset. As shown in the figure, our method exhibits better class separability in the feature space, with samples from different classes clustering more tightly and class boundaries being more distinct. This feature representation capability directly reflects the advantage of our method in classification performance. In contrast, the feature distribution of the ABMIL method is more scattered, with a certain degree of overlap between classes. This visualization result further confirms the effectiveness of our proposed text supervision strategy in enhancing feature representation capability and classification performance.

**FIGURE 6:**
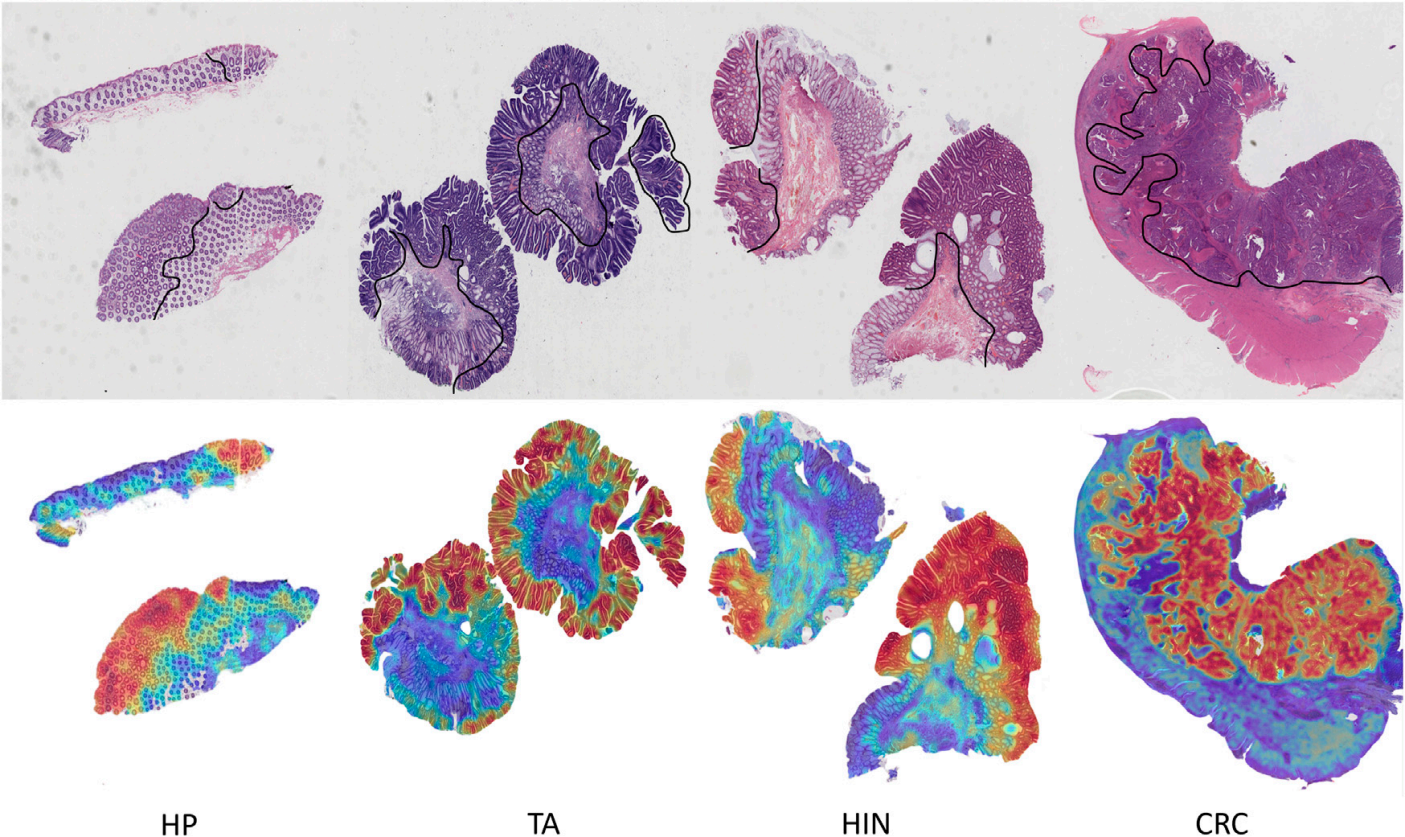
Visualization heatmap results in the colorectal 5-classification dataset.

By visualizing the scores of the corresponding categories in the multiple attention modules onto the patch regions, we can obtain a WSI heatmap of colorectal lesions to demonstrate its interpretability. Figure 6 shows visualized samples of the four abnormal categories in the five-category colorectal dataset. For the CRC category, PAT-MIL can focus on extensive cancerous regions. For the HP, TA, and HIN categories, the model highlights tumor cells and local lesions growing along the wall, which closely aligns with the regions of interest in actual pathological diagnosis.

To gain a deeper understanding of the impact of each component of our proposed text-supervised image classification model on overall performance, we designed and conducted a series of ablation experiments. These experiments included three scenarios: removing the text alignment module, removing the visual module, and removing the text module. The aim was to clarify the specific role of each module in the text-supervised image classification task. All experiments were conducted using the same training set and evaluation criteria to ensure the comparability and reliability of the results.

**FIGURE 7:**
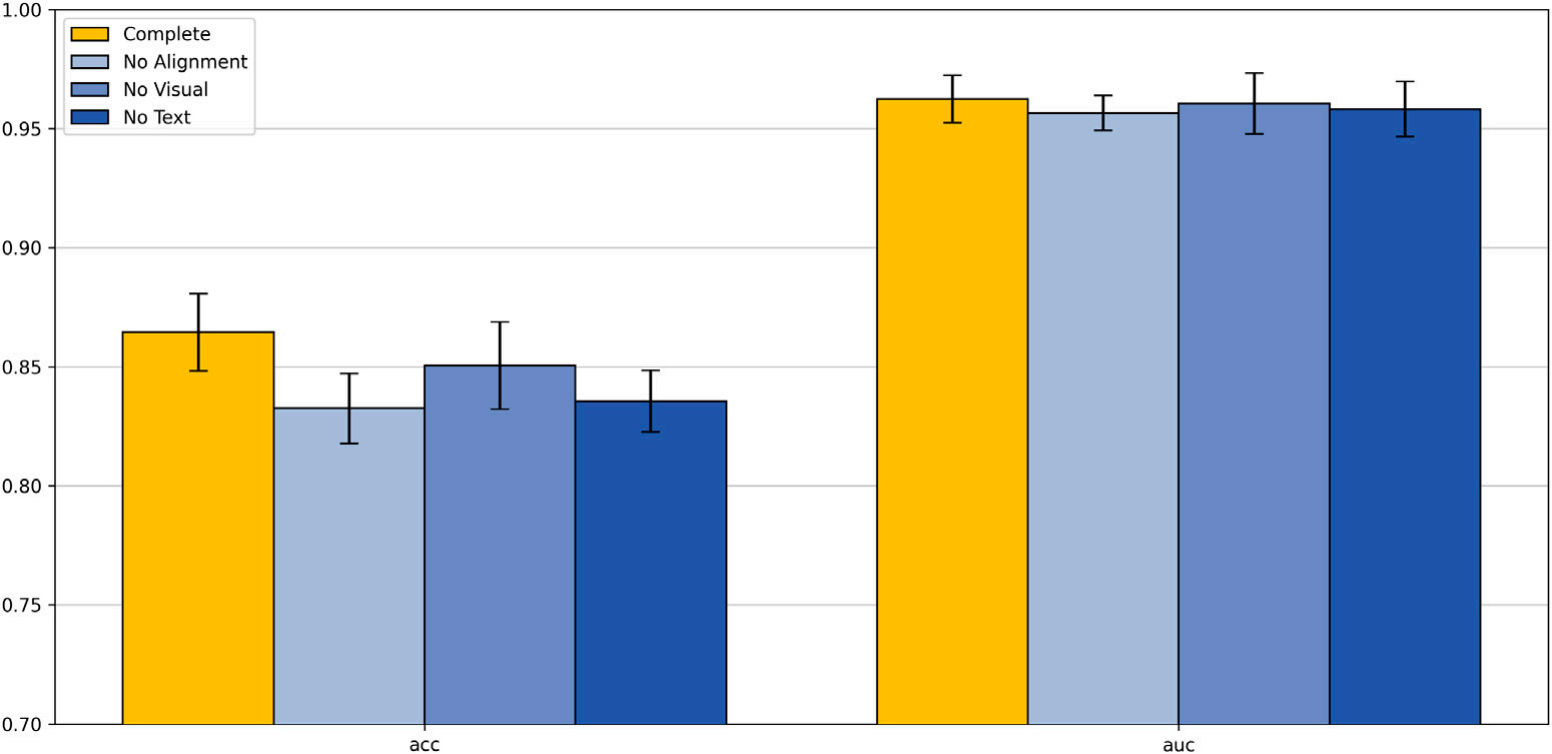
Ablation study.

As shown in Figure 7, removing any module leads to a significant decline in model performance. On the colorectal 5-class dataset, the complete model achieved an accuracy of 86.45% and an AUC of 0.9624, both of which surpass those of other variants. These results clearly demonstrate the critical contribution of each module to the overall performance of the model.

Analyzing these results, we can draw the following conclusions: the removal of the text alignment module highlights the critical role of text-image alignment in multimodal learning; the absence of the visual module indicates that visual features play a central role in classification tasks; the lack of the text module suggests that text features provide important supplementary information to the model. These ablation study results strongly demonstrate that the text alignment, visual module, and text module are the core components of our model, working together to enhance the model’s classification capability. This not only validates the rationality of our model design but also provides valuable insights for further optimization and improvement of multimodal learning models in the future.

## 5 Conclusion and discussion

This study proposes a multimodal deep learning model that combines textual information and WSI for the classification of colorectal pathology images. By introducing pathology expert-optimized text prototypes and an attention mechanism, we effectively aligned visual features with semantic information, significantly enhancing the model’s generalization performance in complex pathological scenarios such as staining inconsistencies and diverse tissue morphologies. Experimental results demonstrate that this method exhibits excellent diagnostic performance in a five-class colorectal pathology classification task, while also reducing reliance on immunohistochemistry experiments, thereby offering the potential to optimize diagnostic processes and reduce medical costs.

Notably, many existing models are primarily evaluated on specific custom datasets, which may limit their ability to generalize to diverse datasets in real-world applications. However, our model demonstrated outstanding generalization performance across different datasets. Specifically, it not only performed excellently on our custom-collected dataset but also achieved remarkable results on the publicly available UniToPatho dataset, achieving an accuracy of 84.1% in a six-class WSI-level task. In contrast, other methods in the literature (Yengec-Tasdemir et al., 2024) achieved an accuracy of 70.3% in a three-class task on the UniToPatho dataset, further highlighting the generalization capability of our model. These results indicate the practical application potential of our model in the classification of colonic adenomatous polyps and lay the foundation for its broader application in clinical settings. These findings validate the proposed model’s ability to generalize across heterogeneous datasets and highlight its potential utility in enhancing diagnostic accuracy and workflow efficiency in clinical pathology.

Despite the encouraging results of this study, its limitations must be acknowledged. The model was trained and validated on retrospective datasets, and future prospective clinical studies are needed to verify its practical effectiveness. Additionally, future research could explore multi-scale feature representation methods to extract more critical information from image patches at different magnifications, further enhancing the model’s robustness and accuracy. Incorporating a wider variety of textual information and multi-center data may also further improve the model’s applicability and diagnostic capability.

Overall, this study demonstrates the potential of multimodal approaches in colorectal pathology classification and provides new solutions for diagnostic tasks of other cancers with significant morphological differences. This method, centered on data efficiency, paves a new path for the practical application of artificial intelligence in pathology and broader medical imaging analysis.

However, one current limitation of our method lies in its reliance on a predefined set of text categories, which may affect flexibility in clinical deployment across different institutions or populations. In future work, we plan to explore adaptive text prototype generation and conduct prospective clinical studies to further validate the model’s practicality and robustness in real-world clinical workflows. Future deployment in clinical settings may require adaptive prototype generation and validation in prospective multicenter studies, especially considering the potential variation in diagnostic terminology and case composition across hospitals.

## Data Availability

The original contributions presented in the study are included in the article/supplementary material, further inquiries can be directed to the corresponding authors.
