# Correction: A pathology-attention multi-instance learning framework for multimodal classification of colorectal lesions

**DOI:** 10.3389/fphar.2025.1666330

**Published:** 2025-07-24

**Authors:** Fanglei Fu, Xuemei Zhang, Zhaoxuan Wang, Luxi Xie, Mingxi Fu, Jing Peng, Jianfeng Wu, Zhe Wang, Tian Guan, Yonghong He, Jin-Shun Lin, Lianghui Zhu, Wenbin Dai

**Affiliations:** ^1^ Department of Life and Health, Shenzhen International Graduate School, Tsinghua University, Shenzhen, Guangdong, China; ^2^ Department of Pathology, Liuzhou People’s Hospital Affiliated to Guangxi Medical University, Liuzhou, Guangxi, China; ^3^ Department of Statistics and Data Science, Washington University in St. Louis, St. Louis, MO, United States; ^4^ State Key Laboratory of Cancer Biology, Department of Pathology, Xijing Hospital and School of Basic Medicine, Fourth Military Medical University, Xi’an, China

**Keywords:** multimodal learning, weakly supervised learning, whole slide image classification, pathology attention, colorectal cancer

In the published article, author “Xuemei Zhang” name was erroneously spelled as “Xeimei Zhang.”

The original version of this article has been updated.

